# Investigation of an MAA Test With Virtual Sound Synthesis

**DOI:** 10.3389/fpsyg.2021.656052

**Published:** 2021-06-02

**Authors:** Ruijie Meng, Jingpeng Xiang, Jinqiu Sang, Chengshi Zheng, Xiaodong Li, Stefan Bleeck, Juanjuan Cai, Jie Wang

**Affiliations:** ^1^Key Laboratory of Noise and Vibration Research, Institute of Acoustics, Chinese Academy of Sciences, Beijing, China; ^2^University of Chinese Academy of Sciences, Beijing, China; ^3^Institute of Sound and Vibration Research, University of Southampton, Hampshire, United Kingdom; ^4^State Key Laboratory of Media Convergence and Communication, Communication University of China, Beijing, China; ^5^School of Electronics and Communication Engineering, Guangzhou University, Guangzhou, China

**Keywords:** localization acuity, the frontal MAA, the lateral MAA, virtual sound synthesis, VBAP

## Abstract

The ability to localize a sound source is very important in our daily life, specifically to analyze auditory scenes in complex acoustic environments. The concept of minimum audible angle (MAA), which is defined as the smallest detectable difference between the incident directions of two sound sources, has been widely used in the research fields of auditory perception to measure localization ability. Measuring MAAs usually involves a reference sound source and either a large number of loudspeakers or a movable sound source in order to reproduce sound sources at a large number of predefined incident directions. However, existing MAA test systems are often cumbersome because they require a large number of loudspeakers or a mechanical rail slide and thus are expensive and inconvenient to use. This study investigates a novel MAA test method using virtual sound source synthesis and avoiding the problems with traditional methods. We compare the perceptual localization acuity of sound sources in two experimental designs: using the virtual presentation and real sound sources. The virtual sound source is reproduced through a pair of loudspeakers weighted by vector-based amplitude panning (VBAP). Results show that the average measured MAA at 0° azimuth is 1.1° and the average measured MAA at 90° azimuth is 3.1° in a virtual acoustic system, meanwhile the average measured MAA at 0° azimuth is about 1.2° and the average measured MAA at 90° azimuth is 3.3° when using the real sound sources. The measurements of the two methods have no significant difference. We conclude that the proposed MAA test system is a suitable alternative to more complicated and expensive setups.

## 1. Introduction

The smallest perceptually detectable difference between the azimuths of two sound sources is called the minimum audible angle (MAA) (Mills, 1958). In 1958, Mills proposed the concept of MAA to measure perceptional auditory spatial acuity and since then, the MAA has been used in many studies on sound localization and auditory perception. For example, the MAA test was used to investigate the precedence effect in sound localization (Litovsky and Macmillan, 1994) or to measure the sound localization acuity of children with cochlear implants (Saberi et al., 1991; Litovsky et al., 2006; Tyler et al., 2010).

Sound source localization is important for auditory scene analysis (McAdams, 1984, 1993; Tyler et al., 2002; Grieco-Calub and Litovsky, 2010; Kerber and Seeber, 2012). There is an increasing demand for affordable and convenient assessment of sound localization ability especially for the hearing impaired and the early identification of hearing loss in children. Often in experimental designs, researchers are restricted to loudspeakers with fixed positions, often with 10° or more separation. It would therefore be preferable to have a controlled method to render virtual stimuli at any angle when measuring the MAA at any desired incident direction.

The MAA measurement method has been conducted in previous researches to measure sound localization acuity with real sound sources (Mills, 1958; Perrott, 1969, 1993; Harris and Sergeant, 1971; Perrott et al., 1989; Saberi et al., 1991; Grantham et al., 2003; Van Deun et al., 2009; Tyler et al., 2010). Various such techniques were developed in the past: Mills (Mills, 1958) used rotating poles to change the incident direction of stimuli in the horizontal plane and the MAA value is about 1°. This apparatus was also popular later in related studies. For example Saberietal (Saberi et al., 1991) used a system of counter-balanced speakers on the pole to measure MAAs in the lateral and dorsal planes. Van Deun et al. (2009) used nine loudspeakers positioned in the frontal horizontal field to measure sound localization, sound lateralization, and binaural masking level differences in young children. Tyler et al. (2010) set up an auditory training system with eight loudspeakers to improve binaural hearing in noise and localization. Perrott (1969, 1993) used 13 loudspeakers in the MAA study with different signal onsets in the horizontal plane and another array with 14 loudspeakers. Harris and Sergeant (1971) set up a track upon which a loudspeaker rode on a little cart, and MAA was computed from the stimulus of Gaussian white noise moving left and right. In Litovsky and Macmillan's experiment (Litovsky and Macmillan, 1994), MAAs were estimated for single noise bursts, and for burst pairs that satisfied the conditions of the precedence effect, but the loudspeakers had to be moved manually between trials. All of these experimental designs based on the real source reproduction are complex, a better design is expected to be applied to clinical utility with easier experiments.

Using a rotating boom method, Mills (1958) measured the MAAs in various directions in the horizontal plane using a two-alternative forced choice procedure. He reported MAAs of about 1°. Similar results were later found by Perrott et al. (1989). For a broadband 0.9 kHz high pass noise, the measured MAA at 0° azimuth is about 1.2° (Perrott, 1993). For broadband noise the measured MAA at 0° azimuth is about 1.6° (Grantham et al., 2003).

Virtual sound synthesis methods were used in studies of virtual reality and artificial sound field generation (McAdams, 2000; Daniel et al., 2010). Existing virtual sound synthesis methods mainly include wave-field synthesis (WFS), Ambisonics, vector-based amplitude panning (VBAP) and binaural synthesis. Wave-field synthesis (WFS) developed by Berkhout et al. (1993) enables the synthesis of sound fields within a rather large listening area. Localization accuracy with wave-field synthesis (WFS) was evaluated using an MAA listening test paradigm (Völk et al., 2012b; Völk, 2016). Ambisonics was firstly proposed by Michael Gerzon as a point source solution for a small listening area and was extended to higher orders of spherical harmonics so that the listening area can be extended significantly (Gerzon, 1977). However, sound reproduction systems through WFS or Ambisonics require tens of loudspeakers. Binaural synthesis (BS) is widely used as a tool aiming at eliciting specific auditory perceptions by means of headphones. An evaluation method was proposed, addressing the binaural synthesis quality by comparing the MAAs measured in the synthesized situation versus the corresponding real situation (Völk et al., 2012a). Völk argued for the use of virtual acoustic methods in psychoacoustics and auditory studies because of their relatively simple application (Völk, 2013). Hohmann discussed the current state and the perspectives of virtual reality technology used in the lab for designing complex audiovisual communication environments for hearing assessment and hearing device design and evaluation, the result showed that the virtual reality lab in its current state marks a step toward more ecological validity in lab-based hearing and hearing device research (Hohmann et al., 2020). Ahrens investigated source localization accuracy with the head mounted displays (HMD) in virtual reality providing a varying amount of visual information, which showed that the lateral localization error induced by wearing HMD was due to alterations of HRTF (Ahrens et al., 2019). However, BS requires individualized head related transfer functions (HRTFs) which are difficult to measure. Berger proposed auditory source localization could be improved for users of generic HRTFs via cross-modal learning (Berger et al., 2018). Pausch employed perceptual tests to evaluate a recently proposed binaural real-time auralization system for hearing aid (HA) users (Pausch and Fels, 2020). But, problems like virtual sound images perceived internalized with binaural synthesis still need to be overcome (Kulkarni and Colburn, 1998). The vector-based amplitude panning (VBAP) was proposed by Pulkki (1997) as stereophonic principles aiming to synthesize an arbitrary sound source between selected pair or triplet of loudspeakers in a plane or in the three-dimension space (Pulkki, 2001a,b; Pulkki and Karjalainen, 2008). Pulkki investigated the localization accuracy of the VBAP method, it was shown that the high-frequency interaural level difference (ILD) cues roughly propose the same directions as low-frequency interaural time difference (ITD) (Pulkki and Karjalainen, 2001). Gröhn (Pulkki, 2001a) conducted a localization accuracy test with VBAP reproduction and non-individualized HRTF reproduction, finding the median value of median azimuth error were 5.6° and 8.3°, the VBAP in this experiment showed the same accuracy as the direct loudspeaker reproduction. The setup of VBAP is relatively simple, however, whether VBAP can be an alternative to conventional methods in hearing research has not been established yet, and some basic perceptual effects such as the MAAs at different reproduction angles should be validated.

In this study, we investigate the feasibility to use the VBAP method to measure the MAAs at 0 ° azimuth and 90° azimuth. This method could reproduce source positions for a single listener at a sweet spot regardless of head rotation. However, the result in sound localization acuity through VBAP is not known yet. This paper first introduces the setup of experiments including the VBAP method and a baseline method. Experiment results are given in section 3, followed by discussions in section 4. Finally, the conclusions are drawn in section 5.

## 2. Materials and Methods

###  Setup

The process of producing the stimuli using VBAP is explained in detail in Pulkki (2001a) and summarized here. For a desired azimuthal incident direction ϕ the signal amplitudes of the selected pair of loudspeakers located at θ_1_, θ_2_ are controlled with gain factors *g*_1_, *g*_2_. The amplitude gains *g*_1_, *g*_2_ are calculated based on Equations (1, 3). Equation (1) calculates the sound amplitudes as a function of incident direction

(1) $$\begin{array}{r} \left(\begin{array}{cc} \cos\theta_{1} & \cos\theta_{2}\\ \sin\theta_{1} & \sin\theta_{2} \end{array}\right)\cdot \left(\begin{array}{c} a_{1}\\ a_{2} \end{array}\right)=\left(\begin{array}{c} \cos\phi \\ \sin\phi \end{array}\right) \end{array}$$

and Equation (2) shows how to calculate the normalized amplitude gains:

(2) $$\begin{array}{r} g_{1}=\frac{a_{1}}{a_{1}^{2}+a_{2}^{2}};g_{2}=\frac{a_{2}}{a_{1}^{2}+a_{2}^{2}} \end{array}$$

**Figure 1:**
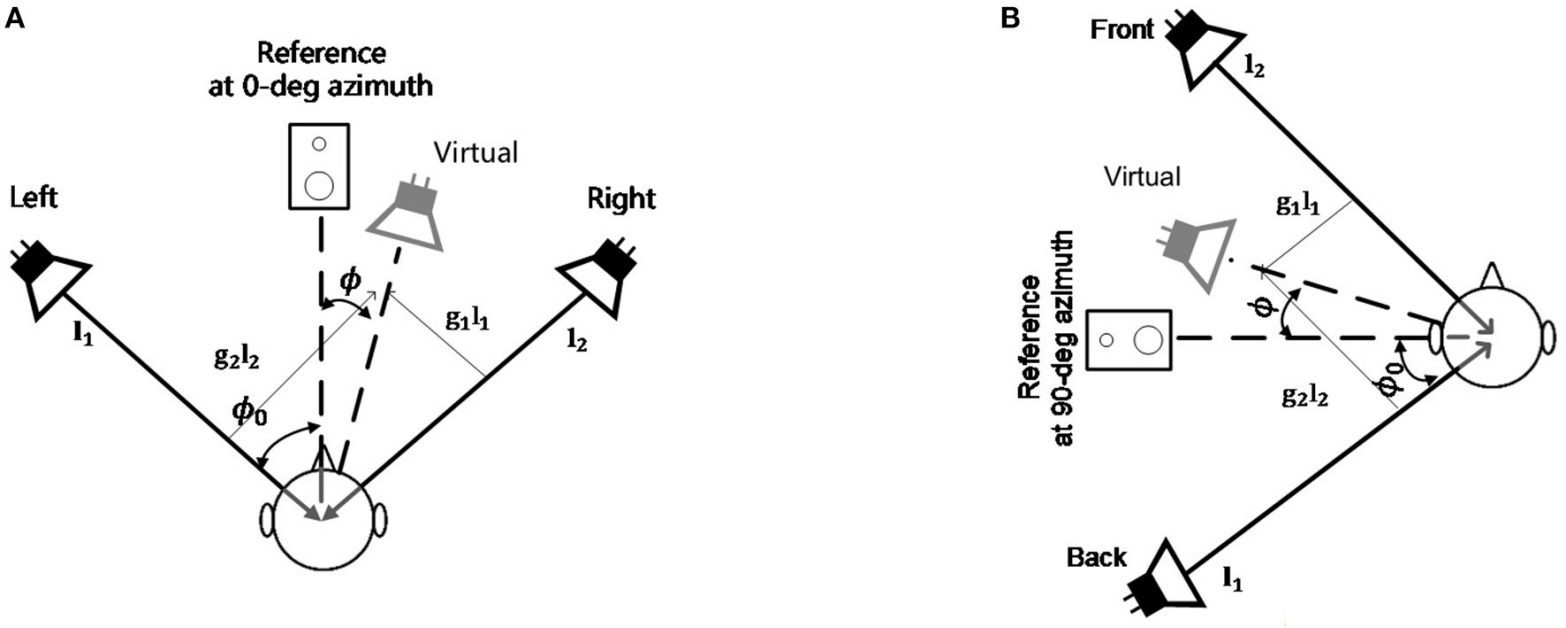
The VBAP system configuration. **(A)** The configuration of the frontal MAA measurement experiment; **(B)** The configuration of the lateral MAA measurement experiment.

In the measurement of the MAA at 0° azimuth (the frontal MAA) with the virtual sound synthesis system, two loudspeakers are located symmetrically with ϕ_0_=30° at each side of the reference as shown in Figure 1A. In the measurement of the MAA at 90° azimuth (the lateral MAA) with the virtual sound synthesis system, two loudspeakers are located symmetrically with ϕ_0_=15° at each side of the reference as shown in Figure 1B. Generally, when the aperture between loudspeakers is wider, the localization accuracy is worse (Pulkki and Karjalainen, 2001). The head rotation can be corrected using the tangent law (Pulkki, 1997):

(3) $$\begin{array}{r} \frac{\tan\phi }{\tan\phi_{0}}=\frac{g_{1}-g_{2}}{g_{1}+g_{2}} \end{array}$$

Theoretically, an accurate synthesis is possible in the horizontal plane by weighting the amplitude gains of the pair of loudspeakers. Therefore, the VBAP method is a promising candidate to provide a simple method of measuring MAA using just two fixed loudspeakers. Sounds were presented at 65 dB(A) with a background level of 28 dB(A), measured with a sound level meter.

###  Participants

Nineteen normal-hearing (thresholds <20 dB HL, measured in a hearing screening test) volunteers participated in the study (aged between 22 and 29). All participants had participated in psychoacoustic localization experiments before and were considered as experienced listeners. A listening room with dimensions of 12.92 m × 6.94 m × 2.67 m (Length × Width × Height) and the reverberation time T60 of 0.1 s was used as the test environment.

###  VBAP Measurement Procedure

Prior to test trials, participants received training to familiarize them with the procedure. The participants were instructed and positioned in a seat 1.8 m away from the loudspeakers in those two experiments. Broadband white noises (0.1–8 kHz) were used as stimuli. The noise stimulus were a train of three 100-ms bursts of Gaussian noise, with a 500-ms silence between bursts. A pair of Bose MusicMonitors were used for sound reproduction through Realtek(R) Audio sound card, in addition, a silent speaker was placed in the middle as a visual reference. Figure 1 showed the frontal and the lateral MAA measurement configuration. In the frontal MAA measurement experiment, the two speakers were placed symmetrically on the left and right of the participants at a fixed angle of 30° in reference to 0° azimuth. In each trial, the stimuli were presented from the right or the left randomly. Participants were instructed to indicate the perceived side of the stimuli in each trial by pointing with their hand toward the right or the left side. The results were recorded by the experimenter seated behind the participant. In the very first trial, the stimuli were presented from 30° (right or left). The initial 30° shift was chosen to ensure that it comfortably exceeded the expected MAAs of all participants. In the lateral MAA measurement experiment, the two speakers were placed symmetrically on the front and back of the participants at a fixed angle of 15° in reference to 90° azimuth. In each trial, the stimuli were presented from the front or the back randomly. Participants were instructed to indicate the perceived side of the stimuli in each trial by pointing with their hand toward the front or the back side. The results were recorded by the experimenter seated behind the participant. In the very first trial, the stimuli were presented from 15° (front or back). This initial 15° shift was chosen to ensure that it comfortably exceeded the expected MAAs of all participants. A 3-down/1-up adaptive procedure (Levitt, 1971) was used to determine the reproduction angle for the next trial, which could be smaller or larger than the previous separation, so as to find the 79.4% correct point on a psychometric function (Schütt et al., 2016). The angular step sizes in the frontal MAA measurement were determined by Parameter Estimation by Sequential Testing (PEST) (Litovsky and Macmillan, 1994), and were: 30°, 15°, 8°, 4°, 2°, 1°, 0.5°. And the angular step sizes in the lateral MAA measurement were: 15°, 8°, 4°, 2°, 1°. The presentation side (left/front or right/back) in each trial was chosen randomly. The experiment ended after six reversals (a reversal is an increase in angle following a decrease, or vice versa), this procedure is converging toward the 79.4% point of the psychometric function. After discarding the first 2 reversals the MAA is defined here as the angular threshold where about 79.4% of all judgments of the relative positions of the sound sources are correct. The average experiment duration for each individual was around 30 min.

###  Baseline Measurement Procedure

**Figure 2:**
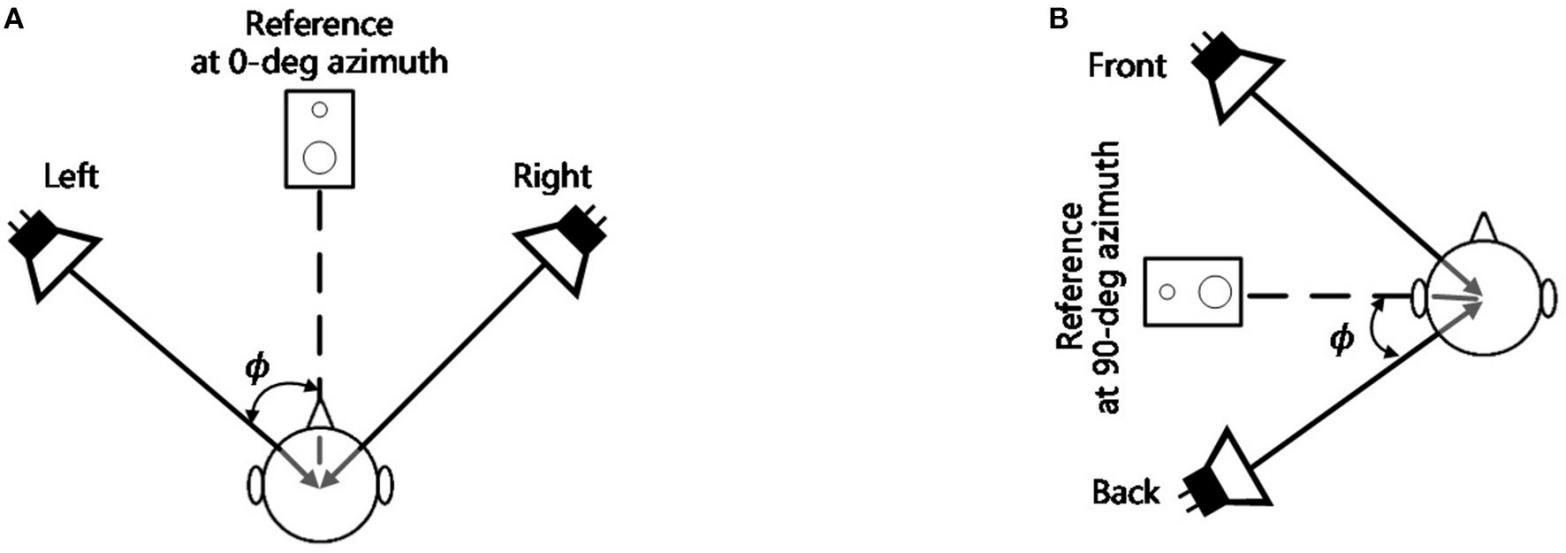
The verification system configuration. **(A)** The configuration of the frontal MAA measurement experiment (0° azimuth), the angle ϕ= ±1°, ±2°, ±4 °, ±8°; **(B)** The configuration of the lateral MAA measurement experiment (90° azimuth), the angle ϕ= ±1°, ±2°, ±4 °, ±8°, ±15°.

In order to verify the accuracy of the VBAP system, an MAA experiment system with the real sources shown in Figure 2 was used as the baseline comparison. In the frontal MAA measurement, this baseline system consisted of a pair of loudspeakers: one was located on the left and the other was located on the right side at either 1°, 2°, 4°, 8° symmetrically in reference to 0° azimuth. In the lateral MAA measurement, this baseline system consisted of a pair of loudspeakers: one was located at the front and the other was located on the back side at either 1°, 2°, 4°, 8°,15° symmetrically in reference to 90° azimuth. The stimuli and the test room were the same as those in the previous VBAP measurement procedure. The participants were instructed and positioned in a seat 1.8 m away from the loudspeakers in those two experiments. When playing the left and right (or the front and back) sound randomly, the participants were answered whether the sound is on the left or the right (front or back). The procedure was conducted twenty times at each angular separation of loudspeakers. The results were averaged and provided a percent correct indicating how often the participants correctly identified the localization. The average experiment duration for each individual was around 30 min.

## 3. Results

**Figure 3:**
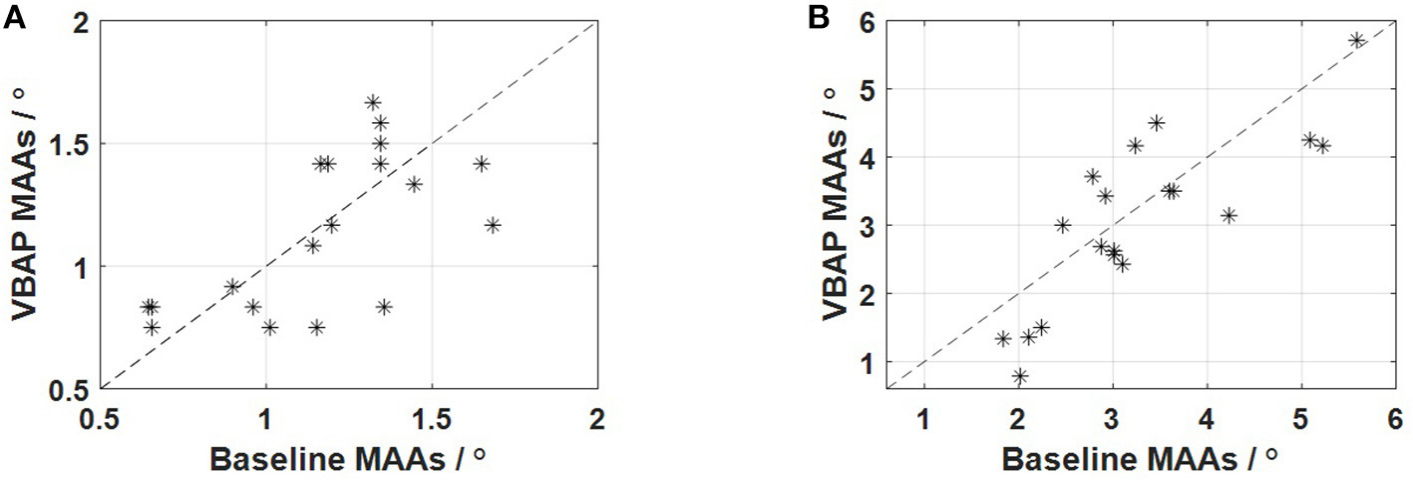
Comparison of the results of the two methods. **(A)** The results of the frontal MAA measurement experiment; **(B)** The results of the lateral MAA measurement experiment.

The average MAAs at 0° azimuth measured by the VBAP method is 1.1° with a range from 0.8° to 1.7° and a standard deviation of 0.3°. The average MAAs at 90° azimuth measured by the VBAP method is 3.1° with a range from 0.8° to 5.7° and a standard deviation of 1.3°. Using the described adaptive method, the MAA is the angle where the psychometric function is 79.4% correct. To establish an equivalent threshold from the baseline method, we employed the following method: percent correct rates were calculated for each angle, and the resulting data were fitted with a psignifit function (Schütt et al., 2016). The percent correct at each angular separation in the baseline method was extracted from subjects' answer data, based on which the fitting curves were used to estimate corresponding MAAs with judgments 79.4% correct. The MAA at 0° azimuth is 1.2° with a range from 0.6° to 1.7° and a standard deviation of 0.3°. The MAA at 90° azimuth is 3.3° with a range from 1.8° to 5.6° and a standard deviation of 1.1°, which is taken as the MAAs from the baseline method. This result is consistent with previous findings that MAA at 0° azimuth is about 1° with a range from 0.7° to 2.5° (Mills, 1958; Perrott, 1969; Harris and Sergeant, 1971; Tyler et al., 2010). In the frontal MAA measurement experiment, we assume that participants have an average of about 50% correct at 0° and almost achieve 100% accuracy at the angular separation of 8°, and in the lateral MAA measurement experiment, we assume that participants have an average of about 50% correct at 0° and achieve 100% accuracy at the angular separation of 15°. The MAA results of the two experiments are shown in Figure 3. Paired *t*-tests of participants' MAAs in both methods were performed to test if there is a significant difference between the baseline data and the VBAP data. As the calculated *p*-values (*t* = 0.43, *p* > 0.05, Cohen's *d* = 0.10 in the frontal experiment; *t* = 1.30, *p* > 0.05, Cohen's *d* = 0.30 in the lateral experiment) are bigger than the p critical value (*p* = 0.05 with 95% confidence), the null hypothesis is accepted, meaning that there is no statistical difference in the same participant's MAA between the VBAP method and the baseline method. For the same participant, we also found that the performance in the frontal MAA measurement experiment is not statistically correlated with the performance in the lateral experiment (*r* = 0.02, *p* > 0.05 in baseline method, *r* = 0.01, *p* > 0.05 in VBAP method). This may mean that people who perform well in the frontal MAA measurement experiment do not necessarily perform well in the lateral MAA measurement experiment.

**Figure 4:**
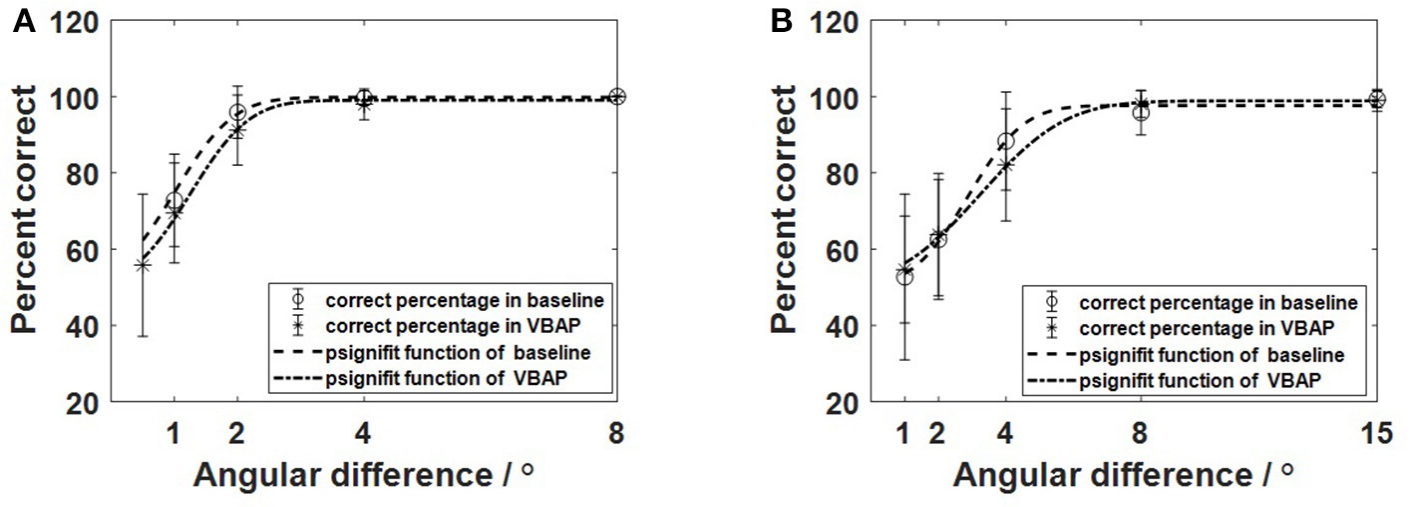
The percentage of correct answers fitted by psignifit function in the baseline (dash line) and the VBAP method (dash-dot line). **(A)** The results of the front MAA measurement experiment; **(B)** The results of the lateral MAA measurement experiment.

To further illustrate the similarity between the baseline method and the VBAP method, we calculated the average percent correct of different angular separation in both experiments (marked star and circle in Figure 4) and fitted curve of the average percent correct of the group at each angle (see Figure 4 dash and dash-dot line). The error bar means variance of the correct percent at each angle in Figure 4. For the frontal experiment, the variance of the deviation between the individual measurement accuracy of each angle is 18.65, 13.11, 9.18, 4.03, and 0%, respectively at 0.5°, 1°, 2°, 4°, 8° in the VBAP method. For the frontal experiment, the variance of the deviation between the individual measurement accuracy of each angle is 12.08, 6.86, 1.91, and 0%, respectively at 1°, 2°, 4°, 8° in the baseline method. For the lateral experiment, the variance of the deviation between the individual measurement accuracy of each angle is 14.01, 16.05, 15.70, 3.48, 2.90%, respectively at 1°, 2°, 4°, 8°, 15° in the VBAP method and 21.74, 15.72, 12.89, 5.84, 2.17%, respectively at 1°, 2°, 4°, 8°, 15° in the basline method. We compared the percent correct of each angle in VBAP method and the baseline method for each individual participant. Paired *t*-tests of participants' results in both methods were performed (ϕ= 1°, *t* = 1.14, *p* > 0.05, Cohen's *d* = 0.26; ϕ= 2°, *t* = 2.25, *p* = 0.04 < 0.05, Cohen's *d* = 0.51; ϕ= 4°, *t* = 1.84, *p* > 0.05, Cohen's *d* = 0.42 in the frontal experiment. ϕ= 1°, *t* = 0.33, *p* > 0.05, Cohen's *d* = 0.08; ϕ= 2°, *t* = 0.34, *p* > 0.05, Cohen's d = 0.08; ϕ= 4°, *t* = 1.44, *p* > 0.05, Cohen's *d* = 0.33; ϕ= 8°, *t* = 1.31, *p* > 0.05, Cohen's *d* = 0.30; ϕ= 15°, *t* = 0.44, *p* > 0.05, Cohen's *d* = 0.10 in the lateral experiment). The above analysis shows that the calculated *p*-values are bigger than the p critical value (0.05 with 95% confidence) except when ϕ is 2° (Cohen's *d* = 0.51, medium effect), which indicates that the two methods are not significantly different to some extent. However, more samples are needed to strongly support the non-significant difference hypothesis.

## 4. Discussion

**Figure 5:**
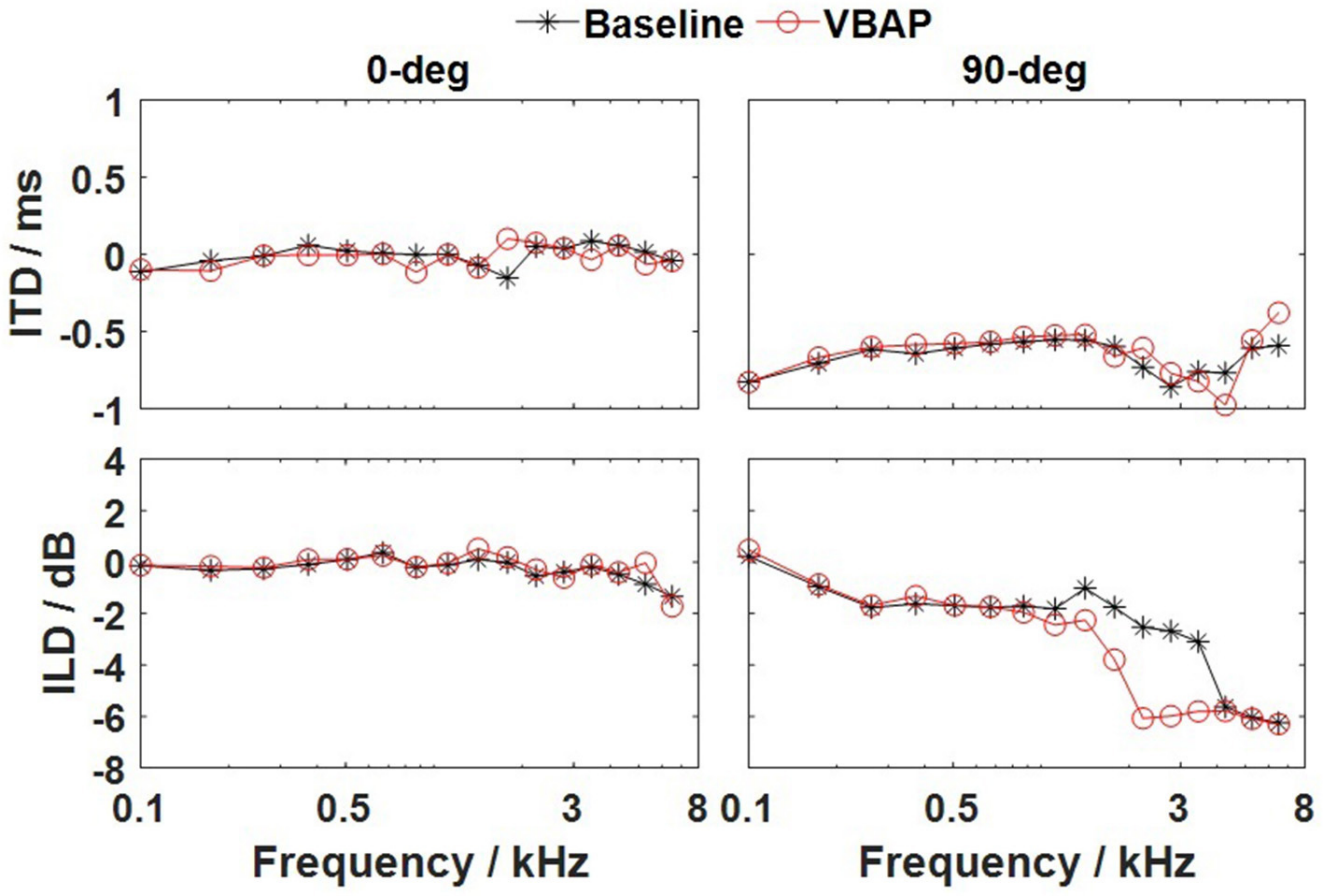
ITDs and ILDs as a function of the critical bands for the baseline method (solid line marked with the black star) and the VBAP method (solid line marked with the red circle). Left column, 0° azimuth of incidence; and the right column, 90° azimuth of incidence. The top and bottom rows show the results for the ITDs and ILDs, respectively.

We compared the MAAs at 0° and 90° azimuth determined in the VBAP method and the measured baseline results, and showed that there is no significant difference in the results obtained by the two methods. To further verify the substitutability of the VBAP method, we conducted acoustic simulations to analyze the binaural cues (ITD and ILD) of the stimuli delivered via VBAP. By convolving the generic non-individualized HRTF of the KEMAR mannequin with the stimuli, the left and right signals are obtained. We divided the stimuli into 16 critical bands with a gammatone filterbank and estimated corresponding ITDs and ILDs through the Binaural Cue Selection Toolbox (Faller and Merimaa, 2004). The simulation results of the ILDs and ITDs in the VBAP method and the baseline method are compared in Figure 5. The left and the right columns show the results for 0-degree azimuth and 90-degree azimuth of incidences, respectively. The top and bottom rows show the results for the ITDs and ILDs, respectively. The VBAP delivers ITDs and ILDs closely consistent with those delivered by the real sound source. Therefore, we conclude that the virtual sound synthesis system is a valid alternative to the conventional apparatus, e.g., a cart runs in the track (Harris and Sergeant, 1971), large scale loudspeaker array (Harris and Sergeant, 1971; Perrott and Saberi, 1990), or a sound boom balanced by weights (Saberi et al., 1991) and can provide a compact and affordable listening test system for measuring MAAs.

This could be useful in the future as an additional tool to diagnose hearing impairment in a clinical setting, and could also be used for the hearing aid fitting process. Due to the principle of rendering virtual sound sources within the angular range between two loudspeakers, a slight misplacement would introduce a large deviation of the incident direction. This position-sensitive attribute is particularly obvious in the hearing tests where small angular differences are required. Improving the localization accuracy of the apparatus as well as the participants' localization accuracy would be beneficial. To reduce the uncertainty of participants' localization, a head-tracking system monitoring the participants' head position would also be useful. However, an appropriate head fixation limiting the head motions is a cheaper option. Moreover, the present sample size is small, and the feasibility of the VBAP method needs to be further verified. These limitations are important issues for our future research, and they are also inevitable problems in clinical applications. Finally, reducing the interval angle between each pair of loudspeakers is likely to provide higher localization acuity in sound source reproduction. However, a large interval angle can provide more virtual sound source locations flexibly without having to move or add loudspeakers. We need to balance between the speaker arrangement flexibility and localization acuity.

## 5. Conclusions

We evaluated the feasibility of a virtual acoustic method to measure MAAs, because conventional apparatuses are usually complicated to use. We used a setup with two loudspeakers driven by sounds based on the vector-based amplitude panning (VBAP) principle. Results show that a resolution around 1° at 0° azimuth and around 3° at 90° azimuth can be achieved by the virtual acoustic test system. To validate the results of MAA test, a baseline measure with real loudspeakers was established with the same participants. The results of “real MAAs” and “virtual MAAs” are not significantly different and thus provide validation of the proposed MAA measurement method.

The virtual acoustic methods provide a convenient and affordable alternative to implement experiments in hearing research and they have the potential for a wider range of applications. For example, assessment of localization skill in hearing-aid fitting and children's localization training in the critical period of auditory development (Harrison et al., 2005). Since the loudspeakers are fixed during the experiment, such methods can be quite convenient for studies involved moving sound sources such as moving minimum audible angle (Hughes and Kearney, 2016).

## Data Availability Statement

The raw data supporting the conclusions of this article will be made available by the authors, without undue reservation.

## Ethics Statement

Written informed consent was obtained from each participant in accordance with the local legislation and institutional requirements.

## Author Contributions

RM: methodology, data analysis, writing the second draft based on the review comments. JW and JC: methodology and modification. SB and CZ: writing-review and editing. XL: writing-review and editing, supervision. JS: methodology, writing-review and editing. JX: methodology, software, formal analysis, and writing-original draft. All authors contributed to the article and approved the submitted version.

## Conflict of Interest

The authors declare that the research was conducted in the absence of any commercial or financial relationships that could be construed as a potential conflict of interest.
